# Designing robot-assisted neurorehabilitation strategies for people with both HIV and stroke

**DOI:** 10.1186/s12984-018-0418-3

**Published:** 2018-08-14

**Authors:** Kevin D. Bui, Michelle J. Johnson

**Affiliations:** 1 Department of Bioengineering, University of Pennsylvania, Philadelphia, USA; 2 Rehabilitation Robotics Lab (a GRASP Lab), University of Pennsylvania, 1800 Lombard Street, Philadelphia, 19146 USA; 3 Department of Physical Medicine and Rehabilitation, Perelman School of Medicine, University of Pennsylvania, Philadelphia, USA

**Keywords:** HIV, Stroke, Neurorehabilitation, Robotics, Developing countries

## Abstract

There is increasing evidence that HIV is an independent risk factor for stroke, resulting in an emerging population of people living with both HIV and stroke all over the world. However, neurorehabilitation strategies for the HIV-stroke population are distinctly lacking, which poses an enormous global health challenge. In order to address this gap, a better understanding of the HIV-stroke population is needed, as well as potential approaches to design effective neurorehabilitation strategies for this population. This review goes into the mechanisms, manifestations, and treatment options of neurologic injury in stroke and HIV, the additional challenges posed by the HIV-stroke population, and rehabilitation engineering approaches for both high and low resource areas. The aim of this review is to connect the underlying neurologic properties in both HIV and stroke to rehabilitation engineering. It reviews what is currently known about the association between HIV and stroke and gaps in current treatment strategies for the HIV-stroke population. We highlight relevant current areas of research that can help advance neurorehabilitation strategies specifically for the HIV-stroke population. We then explore how robot-assisted rehabilitation combined with community-based rehabilitation could be used as a potential approach to meet the challenges posed by the HIV-stroke population. We include some of our own work exploring a community-based robotic rehabilitation exercise system. The most relevant strategies will be ones that not only take into account the individual status of the patient but also the cultural and economic considerations of their respective environment.

## Background

Stroke is a leading cause of death and disability in high income countries while both stroke and human immunodeficiency virus (HIV) are leading causes of death and disability in lower income countries [1, 2]. There is increasing evidence that HIV is an independent risk factor for stroke, resulting in an emerging population of people living with both HIV and stroke all over the world in both high and low resource areas [3–9]. Little research has been conducted on this population, particularly from a neurorehabilitation standpoint. It is important to consider the HIV-stroke population from this viewpoint because both are chronic diseases associated with lasting neurologic injury and require extensive amounts of monitoring, assessment, and treatment. While dealing with one is difficult enough, the added burden on the patient, their family, and health care providers from both diseases is an impending global health challenge that must be addressed.

Studies to date looking into the relationship between HIV and stroke have taken an epidemiological or pathophysiological approach, both confirming and trying to understand the cause for increased stroke rates in the HIV population [3–11]. However, very little is being done to address the physical, cognitive, social, and other problems that the HIV-stroke population currently faces. There is a need to develop relevant evidence-driven neurorehabilitation strategies for the HIV-stroke population to address the gaps in care and improve outcomes related to quality of life. This is an issue that is globally relevant given the rapidly aging HIV population in high income countries (HICs) and the increasing stroke rates in low and middle income countries (LMICs), where HIV is more prevalent [12]. Developing these solutions can also lead to advancements that may benefit people with just stroke, just HIV, and other populations dealing with multiple comorbidities.

As outlined by the National Institutes of Health, improving prevention or treatment of HIV-associated comorbidities and complications has become a high priority area in HIV/AIDS-related research [13]. Additionally, in the context of LMICs, the World Health Organization (WHO) has said that addressing the disability issue “is a development priority because of the higher prevalence of disability in lower-income countries and because disability and poverty reinforce and perpetuate one another” [14]. This review approaches the HIV-stroke population from a neurorehabilitation viewpoint — a viewpoint that is currently lacking for this population. Neurorehabilitation refers to the concept of intentionally affecting recovery in the nervous system through targeted rehabilitation exercises that can span across physical, cognitive, psychological, social, and cultural domains. To successfully develop neurorehabilitation strategies for the HIV-stroke population, a thorough understanding of multiple areas is needed, ranging from the molecular to the behavioral to the engineering. This includes the mechanisms, manifestations, and treatment options of neurologic injury in stroke and HIV, the additional challenges posed by the HIV-stroke population, and rehabilitation engineering approaches for both high and low resource areas.

This review also goes into strategies for developing robot-based neurorehabilitation strategies. Robot-assisted technologies have shown to be a promising approach in rehabilitation with the emergence of the rehabilitation robotics field. We explore how robot-assisted rehabilitation could be used as a potential approach to designing neurorehabilitation strategies for the HIV-stroke population. We highlight relevant areas of research in the field of rehabilitation robotics that can help advance research on the HIV-stroke population such as robot-based biomarkers of motor impairment, motor learning, cognitive assessment and rehabilitation, and affordable rehabilitation robotics. Other rehabilitation techniques, such as community-based rehabilitation, also have utility in designing new neurorehabilitation strategies. We detail a system we have built that combines both robotic and community-based rehabilitation — the Rehabilitation Community-Based Affordable Robot Exercise System (Rehab CARES) Gym — and is specifically designed to be deployed in LMICs that can be used as a way to provide neurorehabilitation to the HIV-stroke population.

The aim of this review is to connect the underlying neurologic properties in both HIV and stroke to rehabilitation engineering. By doing so, we hope to highlight both the gaps in research in order to spur the development of novel neurorehabilitation approaches for the HIV-stroke population and the opportunities to expand the scope of the rehabilitation robotics field.

## Neurologic injury in stroke

Stroke affects 800,000 people in the United States each year, costing roughly 34 billion USD in health care services, medications, and lost productivity [1]. It is the fifth leading cause of death and a leading cause of disability [1]. In LMICs, stroke rates have increased by 100 percent from 2002-2012 [12]. The heterogeneous nature of stroke poses a challenge in developing effective solutions that are applicable to the spectrum of stroke outcomes. Many of the advancements in the field have not yet made it to LMICs. As such, making these solutions accessible in a global context poses an additional unmet need.

Stroke is a neurologic disease resulting from either a blockage in a blood vessel supplying the brain or a rupture of a blood vessel in the brain, termed an ischemic or a hemorrhagic stroke, respectively. Standard stroke risk factors include high blood pressure, high cholesterol, diabetes mellitus, sedentary lifestyle, and smoking [15]. Because these factors are addressable, stroke is seen as a preventable disease. Depending on factors such as the size and location of the brain lesion resulting from the stroke, varying degrees of cognitive and motor impairment can result in difficulties performing activities of daily living and significantly reduce the quality of life for the stroke patient. These life-altering impairments can manifest as physical impairment — such as hemiparesis, muscle weakness, and spasticity — or cognitive impairment — such as vision problems, memory loss, aphasia, and other issues.

### Functional brain changes after stroke

Functional changes in the brain have been extensively studied in various magnetic resonance imaging (MRI) studies, and provide a glimpse into the changes that happen in the brain on a systems level. Functional changes refer to how regions of the brain activate differently after a stroke in a resting state or during the performance of a task. Functional MRI (fMRI) studies have demonstrated that the brain can respond in several different ways following stroke. Ward showed that in stroke patients who had intact primary motor cortices, more complete recovery was achieved when brain activation patterns mirrored that of healthy controls, while those with poorer recovery recruited additional motor-related regions in the brain [16]. A negative correlation was shown between outcome and task-related activation in regions associated with motor movement [16]. Bilateral and contralesional recruitment can occur involving the contralesional (the side of the brain not affected by the lesion) and ipsilesional (the side of the brain with the stroke lesion) parts of the supplementary motor area, cingulate motor areas, premotor cortex, posterior parietal cortex, and cerebellum [16]. Non-motor brain regions can be involved in motor recovery as well [16, 17]. Other longitudinal fMRI studies have shown similar results as Ward, with a consistent pattern of initial contralesional recruitment, with recovery dependent on how much activity is restored to the ipsilesional side [18–20].

### Structural changes after stroke

Structural changes have been examined using diffusion tensor imaging, which visualizes the structural integrity of white matter tracts in the brain by measuring the diffusion of water across these tracts [21]. Damage to the white matter results in reduced anisotropic diffusion, and this can be quantified in values such as fractional anisotropy (FA) and mean diffusivity [21]. Warach used diffusion-weighted imaging to measure changes in apparent diffusion coefficients (ADCs), showing that these values decreased after stroke and slowly returned toward normal values in the chronic phase of stroke, allowing for acute lesions adjacent to chronic infarcts to be readily distinguished [22]. Others have shown that the infarct regions in the ipsilateral descending corticospinal tract of stroke patients resulted in lower FA values [23].

### Stroke treatment

#### Acute phase

The acute phase of stroke refers to the first month after stroke onset. There are limited treatment options at the time of stroke onset. An ischemic stroke can be treated with tissue plasminogen activator (TPA) — a protein that dissolves blood clots — if it is administered within four and a half hours of stroke onset [24]. The clot can also be mechanically removed. These treatments can reduce the long term effects but do not guarantee full recovery given the short time window for success. Full recovery is associated with a return to pre-stroke neurologic and functional conditions. On the neurologic side, recovery involves reversal of diaschisis, neurogenesis and repair, and alteration of existing pathways [25, 26]. Functional recovery is closely intertwined with neurologic recovery, as changes in physical or cognitive ability are reflective of changes in brain function.

### Spontaneous vs use-dependent recovery

#### Sub-acute phase

Despite the limited window for administering TPA, recovery can still occur after stroke. Recovery can generally be broken in two separate categories — spontaneous and use-dependent recovery. Spontaneous recovery is the period of time shortly following a stroke when the brain naturally compensates for lost function by forming new neurons or recruiting other parts of the brain to execute a damaged function and is thus in a state of increased neuroplasticity [27]. Neurogenesis can occur in the affected brain region following a stroke, and the more neurogenesis there is, the more function is recovered [28]. However, the specific mechanisms of neurogenesis or how to increase the output remains an open question.

The time between the first month and sixth month after stroke onset is referred to as the sub-acute phase. The majority of regained function occurs in the first three months following a stroke and spontaneous recovery plateaus after six months [29]. The ability of a patient to naturally recover function is dependent on the severity and location of the stroke — mild and moderate stroke patients more so than severe stroke patients have much greater chances of recovering or improving function [29].

#### Chronic phase

Use-dependent recovery can serve two purposes. It aims to help the patient regain additional function more quickly than spontaneous recovery alone and can also promote recovery in the chronic phase of stroke, which is beyond six months after stroke onset. Use-dependent therapy promotes additional neuroplasticity to improve recovery through repetition [30–32]. Intensive use-dependent therapy during the acute phase of stroke leverages the period of spontaneous neuroplasticity to maximize recovery outcomes. Inducing additional recovery in the chronic phase — beyond six months after the onset of stroke — is a key focus in rehabilitation, as 55-75 percent of stroke patients experience lasting upper limb impairment [33].

### Rehabilitation strategies

Understanding the functional and structural changes that result from a stroke and how these progress over the course of recovery is important in order to design neurorehabilitation strategies that can drive brain reorganization and functional recovery in a targeted manner. Conventional treatment after a stroke involves a combination of physical, cognitive, occupational, and speech therapy, among other forms of support. These therapies require dedicated rehabilitation professionals, and data show that the burden of stroke management will continue to increase [34]. Combined with current trends reflecting a shortage of physical therapists in the U.S. workforce, quality and access to care will be significantly impacted [35]. This pressure is already felt in LMICs [14]. Even some regions in HICs, particularly rural areas, are already experiencing the effects of a shortage on rehabilitation professionals and services [36].

The need for innovative stroke rehabilitation strategies is a global need. As a result, a lot of research has gone into developing more effective rehabilitation strategies that can augment the abilities of therapists and bring rehabilitation services to more patients. Going hand in hand with the need for innovative stroke rehabilitation strategies is the need for reliable, quantitative ways to assess and measure progress. Clinical assessments are useful for providing insight on the overall status of the patient, but the distinction between different groups is often very coarse (i.e. severe vs. moderate vs. low impairment or dementia vs. no dementia). Current assessments include motor function tests such as the Fugl-Meyer Test, Wolf Motor Function Test, Nine-hole Pegboard Test, Grooved Pegboard Test, Box and Blocks Test, Timed Up and Go Test, Ten Meter Walk, and Modified Ashworth Scale; cognitive tests such as the Montreal Cognitive Assessment, Trail Making Tests, and Mini-Mental State Examination; and overall neurologic examinations such as the National Institutes of Health Stroke Scale, the Barthel Index, Functional Independence Measure, and the Modified Rankin Scale [37–50]. The ideal clinical test would be quick, easy to use, reliable, and responsive to meaningful clinical change, but no test currently meets all the criteria [51].

There are multiple limitations to current clinical tests. They often require a trained professional and take time to administer. In addition, the set of clinical tests that are administered can vary by the resources and time available. Even when the same test is administered, results can vary depending on who is administering the test, thus limiting the ability to identify milder changes. As such, even though there are established assessments and rehabilitation strategies, there is a lot of room for improving these areas with innovative approaches.

## Neurologic injury in HIV

Each year, 40,000 new people are diagnosed with HIV, and 1.2 million people live with HIV in the U.S. [52]. There are 36.7 million people living with HIV worldwide, with the majority living in LMICs [2]. HIV is an incurable disease that attacks the body’s T cells, which if left untreated, leads to acquired immune deficiency syndrome (AIDS) and opportunistic diseases infecting the body. With the advent of antiretroviral therapy (ART), HIV has changed from a life-threatening to a chronic disease, resulting in a rapidly aging HIV population. By 2020, half of the HIV population in the U.S. will be over 50 years old [53]. ART has been a life-changing development, but there remain problems that have yet to be addressed, namely the prevalence of neurocognitive disorders, impairments, activity limitations, and disability.

### HIV-associated neurocognitive disorders

HIV-associated neurocognitive disorders (HAND) are a set of neurologic disorders of varying severity that affect cognitive, motor, and behavioral domains [54]. The categories of HAND, as defined by the Frascati criteria, include asymptomatic neurocognitive impairment (ANI), minor neurocognitive disorder (MND), and HIV-associated dementia (HAD) [55]. ART has decreased the incidence of HAD, while the incidence and prevalence of milder forms of HAND remains high at about 40 percent of the HIV population [56, 57]. HAND can impact the quality of life of a patient by contributing to HIV-associated disability and interfering with their ability to independently perform activities of daily living, such as adhering to medication, leading to more serious downstream problems [54, 58]. When the Frascati criteria was established, minor cognitive-motor disorder was encompassed into MND and motor-related assessments were minimized for the most part. However, HAND can also impact physical domains as well, leading to neuropathy, slowed movement, ataxia, impaired gait, and diminished fine motor skills [59].

The gold standard for diagnosing HAND is by an extensive neuropsychological battery that assesses a patient’s information processing, learning and memory, executive function, verbal fluency, working memory, and motor domains [55]. This requires a trained professional and is a time-consuming process. In settings where an in-depth assessment cannot be administered, brief screening tests are desired [55]. The most commonly used screening test is the International HIV Dementia Scale (IHDS) [60]. Other screening tests include the HIV Dementia Scale (HDS) and Montreal Cognitive Assessment (MoCA), but neither performs well in distinguishing the milder forms of HAND [61]. Motor impairment is not extensively tested in these assessments, but may have utility in diagnosing neurocognitive disorders when normative data is not available [62].

### Pathophysiology of HIV-associated neurocognitive disorders

The prevalence of HAND likely remains high because current ART regimens are not successfully penetrating the central nervous system [56]. The most widely accepted model states that HIV invades the brain via a “Trojan Horse” method in which infected monocytes cross the blood-brain barrier and differentiate into macrophages [63, 64]. This then leads to neurodegeneration and the symptoms seen in HAND. The neurodegeneration is caused from chronic neuroinflammation resulting from a combination of cytokine and chemokine effects, excitotoxicity, or oxidative stress [57]. This in turn leads to synaptic disruption and impaired neurogenesis. While these issues may be addressed by developing different drug therapies that are better able to cross the blood-brain barrier and target the mechanisms of neurodegeneration, other approaches should be considered to manage the symptoms. There is also emerging research suggesting that ART itself could have neurotoxic effects on the brain, leading to the production of compounds similar to those seen in Alzheimer’s disease [57].

### Functional brain changes after HIV infection

Much like stroke, the effects of HIV on the central nervous system have been observed using MRI methods. The changes in the brain due to HIV are visible even before HAND can be clinically diagnosed [65]. Fronto-striatal circuits have been shown to be altered by HIV, with the left inferior frontal gyrus and left caudate being the most commonly affected regions [65–68]. Studies have shown that HIV also impacts complex information processing and selective attention, establishing a connection between the affected fronto-striatal circuits and observable behavior [68]. Melrose demonstrated that functional changes in the prefrontal cortex and basal ganglia, which are associated with working memory, occur before structural changes [67, 69]. Neurologic changes can result in minor cognitive or motor disorders and progress to more severe dementia if the HIV is left untreated [70]. Other neurologic effects of HIV include increased activation in the lateral prefrontal cortex and delayed motor learning in HIV-infected children [65, 71].

### Structural brain changes after HIV infection

Structurally, HIV results in cortical thinning in primary sensorimotor, premotor, and visual areas, with prefrontal and parietal tissue loss showing a correlation with slowing of psychomotor speed [72]. Volume loss in the striatal, hippocampal, and white matter areas has been shown to begin in the asymptomatic stages of HAND [73]. Studies have shown that people with HIV had significant reductions in brain volumetrics in the amygdala, caudate, corpus callosum, and putamen despite ART treatment [74, 75]. These findings were independent of aging, which can also increase the vulnerability of the brain. Changes in brain structure have been shown to occur within a year of HIV infection [76]. Another study showed that gray matter decreases in the anterior cingulate and temporal cortices along with white matter reduction in the midbrain region were associated with cognitive decline, while motor dysfunction was associated with basal ganglia gray matter atrophy [77]. These structural changes and the prevalence of HAND demonstrate that while HIV can be well controlled by ART, there are still detrimental effects of HIV that have yet to be addressed.

### Rehabilitation strategies for the HIV population

In a Canada-based study, upwards of 80 percent of Canadians living with HIV reported dealing with an impairment, activity limitation, or social participation restriction [78]. Another study in South Africa on over 1,000 people living with HIV showed that more than a third experience the onset of disability [58]. HIV can accelerate the aging process and lead to frailty and physical impairment earlier on in life [79]. Thus, rehabilitation strategies must address both the cognitive and physical impairments resulting from HIV.

Physical impairments resulting from HIV include chronic pain, joint stiffness, and muscle weakness [59]. However, the number of HIV patients receiving physical therapy is much lower than the number who report dealing with physical limitations [80]. In addition, the fluctuating, episodic nature of HIV can pose additional complications in the day-to-day performance of the patient [81]. Episodic disability is defined as periods of good health interrupted by potentially debilitating periods of disability. This can lead to fluctuations in performance on both short and long timescales over the course of living with HIV and can impact activities of daily living or the ability to hold a job, making occupational therapy useful for the HIV population [82]. These periods of disability can manifest either from HIV or the treatment itself.

The call for rehabilitation strategies specific to the HIV population has been a relatively recent development by developed countries, but it is a need that is magnified in LMICs. Stroke neurorehabilitation strategies have received far greater focus while there is a paucity of neurorehabilitation successes in HIV populations, who are in dire need of such strategies. Rehabilitation in HIV consists of activities and services that address these restrictions while taking into account the distinct physiological, emotional, and societal features of HIV [81]. Within the framework of rehabilitation for people living with HIV, ensuring a wide selection of traditional and specialized professionals (i.e. physical and occupational therapists), services (i.e. AIDS service organizations and alternative therapists), and support (i.e. community workers, legal counselors, social support groups) is a key focus [81]. Despite the existence of a rehabilitation framework, people living with HIV still struggle to gain access to the rehabilitation services they need, often from a lack of awareness on both the patient and care provider side [83]. A challenge in HIV and rehabilitation is the increasing presence of comorbidities — such as diabetes, Hepatitus C, cardiovascular disease, renal disease, and frailty — that can complicate already existing disabilities [79, 84].

A first step in addressing the need is increasing awareness among health care professionals to facilitate access to rehabilitation services for people with HIV, as few rehabilitation professionals knowingly work with someone living with HIV [81]. This indicates a gap in service and a need for HIV-specific training and guidance. Another necessary step is a concerted effort to assess the effectiveness of rehabilitation services [85]. A method for developing clinical practice guidelines in HIV rehabilitation has been proposed by O’Brien and colleagues, focused on understanding the diversity of people living with HIV, taking a client-centered and holistic approach, and maximizing access to rehabilitation services [85]. These guidelines or a similar approach can inform HIV rehabilitation practices that are evidence-based, practical, and accessible. To achieve this, it has been suggested that research in HIV rehabilitation should focus on access to rehabilitation and models of rehabilitation service provision such as early screening and assessment for disability to identify the need for rehabilitation, understanding the transition throughout the HIV continuum of care, and tailoring service delivery to increase the accessibility of rehabilitation to different populations [85].

## Stroke in the HIV population

The life expectancy of someone living with HIV in the United States has increased from under 40 years in 1996 to 73.1 years in 2011 [86]. While it is still below the general population’s life expectancy of 78.8 years, the increased lifespan naturally exposes the HIV population to conventional stroke risk factors [87]. This means that the presentation of both HIV and stroke in a patient can sometimes be coincidental.

However, there is a body of research using epidemiological and pathophysiological methods establishing an association between HIV and stroke [3–11]. Several possible explanations for why HIV causes an increased risk of stroke have been hypothesized. These include opportunistic infection, HIV-associated vasculopathy, cardioembolism, chronic inflammation, and the neurotoxicity of ART itself [3]. A study on the Veterans Aging Cohort, consisting of 76,835 male veterans, showed that HIV infection is associated with an increased ischemic stroke risk among HIV-infected compared with demographically and behaviorally similar uninfected male veterans [88]. In 2012, Chow reported that stroke rates were higher in the HIV population — particularly in young patients and women — independent of typical stroke risk factors compared to the general population in a Boston healthcare system [5]. From 1997-2006, there was a 60 percent increase in stroke rates in the U.S. HIV population despite stroke rates in the general population decreasing by seven percent [4]. Combined with increasing stroke rates in LMICs where HIV is more prevalent, the HIV-stroke population is one that is emerging in both HICs and LMICs [12].

In the U.S., the mean age of patients with HIV at the time of their first stroke was 48.4 years old as of 2006, up from 42.9 years of age in 1997 since the introduction of ART [4]. This is considerably lower than the average age of stroke onset of the general population, which is 70.7 years of age [1]. In a recent study in a U.S. HIV population, the incidence of cerebrovascular event — defined as ischemic stroke, hemmoraghic stroke, and transient ischemic attack — was 3.87 per 1000 years lived [89]. Another study found the incidence of just ischemic stroke to be 1.25 per 1000 years lived [90]. Compared to the HIV-stroke population in the U.S., the HIV-stroke population in areas such as Sub-Saharan Africa is considerably younger. Two studies in South Africa and Malawi showed that the mean age of stroke in HIV patients was 33.4 and 39.8 years old, respectively [7, 8]. Besides the lower age of stroke in HIV patients compared to the U.S., it is also important to note that these particular HIV-stroke patients did not present with typical risk factors of stroke. The HIV prevalence in these countries is 11 and 12 percent of the total population, compared to under 0.5 percent in the U.S. [7, 8]. In some reported cases in LMICs, stroke was the presenting factor that led to HIV diagnosis [7].

### Treatment strategies

Current treatment strategies for people with both HIV and stroke often do not account for the presence of both diseases. For example, the HIV status of someone who has suffered a stroke is usually not a factor when administering treatment or therapy. In other cases, stroke can be the initial manifestation of HIV [7, 91]. In addition, emergency rooms are often not equipped for real-time HIV testing [9]. The effects of drug interactions on the patient remain unknown and thus warrant further investigation. Efforts toward reducing the neurotoxicity of ART, making the central nervous system more permeable to ART to limit chronic neuroinflammation, and finding a cure to HIV are long-term, high-priority goals that will help the treatment and management of the HIV-stroke population [13]. However, these do not benefit the current population living with the challenges of both conditions, and there is a distinct lack of rehabilitation strategies specific to the HIV-stroke population. This is an important need because ignoring the episodic nature and associated comorbidities of HIV during stroke recovery could affect outcomes in ways that are not seen in the stroke population [82]. While HIV alone may present with deficits that necessitate rehabilitation, the occurrence of both HIV and stroke is different from other comorbidities that may show up in either HIV or stroke alone because both result in neurological damage. Thus, this necessitates a treatment approach that has not yet been implemented that accounts for both HIV and stroke.

### Challenges

There are various challenges that should be considered when coming up with rehabilitation and treatment strategies for the HIV-stroke population. The presence of HIV prior to stroke can alter the approach to managing a person with both HIV and stroke. These challenges — while not necessarily exclusive to the HIV-stroke population but are certainly magnified — include joint cognitive and motor deficits, lack of uniform clinical assessments, unknown changes in the brain, psychosocial issues, and accessibility to services.

#### Joint cognitive and motor deficits

The presentation of motor and cognitive deficits could be much more varied in the HIV-stroke population. Factors such as the severity of HAND compounded with the stroke lesion location and size means that the presentation of deficits spanning both motor and cognitive domains could require additional management strategies compared to the HIV population or stroke population alone. For example, compared to a patient with just stroke, someone with HAND who suffered a stroke confined to the primary motor cortex would have the neurocognitive deficits associated with HAND on top of the motor impairment from the stroke. The increased variability across the spectrum of combined cognitive and motor impairments could introduce added complexity in both assessment and treatment of the patient. Many current motor rehabilitation strategies do not take into account the effects cognitive impairment can have on overall recovery [92–94]. More research needs to be done to shed light on this area as cognitive impairment can be an important factor in choosing the most effective motor recovery intervention [92–95]. The connection between cognitive and motor function can be seen in HIV, where studies have shown that cognitive function can improve from aerobic or strength resistance activity [96]. Thus, the presence of both cognitive and motor deficits is a challenge that must be addressed in order to develop effective neurorehabilitation strategies.

#### Lack of uniform clinical assessments

HIV and stroke have their own sets of clinical tests to assess motor, cognitive, and other domains. One of the few studies looking at both the HIV and stroke populations establishes a measure of fatigue across HIV, stroke, and cancer [97]. The lack of uniform clinical tests makes it more difficult to assess the HIV-stroke population, and there are no established ways to account for the presence of the other disease during assessment. For example, a pen-and-paper cognitive test during a neuropsychological assessment for HAND would be difficult for someone who suffered a stroke resulting in hemiparesis of their dominant hand. This could also apply to a stroke patient without HIV presenting with neurocognitive deficits. In addition, the results could be misrepresented even if the patient were able to complete the task with their non-dominant hand. Certain tests require data from a large healthy population in order to normalize the scores, which could vary by country and could be affected by cultural factors, such as the Trail Making Tests [98]. On top of this, a number of other coinfections and comorbidities such as diabetes, bone and muscle dysfunction, and age-related frailty can impact the management of the patient and the ability to perform assessments.

#### Unknown structural and functional changes in the brain

It is unknown how the presence of HIV and stroke jointly affects the functional and structural properties of the brain. As discussed earlier, both diseases independently result in neurologic changes [16–23, 65–77]. To date, comorbidities that may have an effect on neurologic properties have often been criteria for exclusion in imaging studies, thus imaging data on the HIV-stroke population is lacking. However, the presence of HIV could be priming the brain prior to the onset of stroke and could have various implications that are still unknown. Given the advances in imaging technologies and analytical methods, there is the opportunity for useful knowledge regarding the combined neurologic effects of HIV and stroke to emerge that can drive the development of neurorehabilitation strategies.

#### Psychosocial issues

Psychosocial issues resulting from both stroke and HIV can pose a challenge in effectively reaching those who would benefit from a targeted rehabilitation strategy [99–101]. Because of the stigma associated with HIV in various countries, seeking care or revealing one’s HIV status can be a daunting prospect [102]. The psychological effects of living with both HIV and stroke should be taken into account and may require additional considerations when designing treatment regimens.

#### Accessibility

There is a lack of health care professionals who are familiar with the needs of both the HIV and stroke populations and the available resources. This challenge is magnified in LMICs, where an increasing double burden exists of malnutrition and infectious diseases with new problems such as chronic conditions [14]. These resource challenges can also be seen in some areas in HICs, particularly rural areas where it is harder to access the necessary care [36]. In both these areas, the supply chain for rehabilitation services may not be effective or adequate in reaching a lot of people.

## Designing robot-assisted neurorehabilitation strategies for HIV and stroke

The ideal solution to the challenges posed by the HIV-stroke population would be one that is applicable across the combined spectrum of cognitive, motor, and social impairments. On top of that, it should be scalable and accessible to the HIV-stroke populations not only in the U.S. and other high resource areas but also in lower resource areas around the world. While no such solution currently exists, there are potential approaches that can be leveraged.

As highlighted earlier, one of the biggest challenges with the HIV-stroke population is the increased prevalence of joint cognitive and motor deficits. While this challenge is magnified in the HIV-stroke population, this is not a challenge that is unique to this population, as many neurologic diseases can result in some combination of cognitive and motor deficits. Advanced assistive and rehabilitation technologies, namely robot-based methods, provide an approach to assess impairment and provide rehabilitation that can address many of the challenges faced with the HIV-stroke population [103]. Rehabilitation robotics has demonstrated the ability to be at least as effective as high-intensity physical therapy [104, 105]. The upside that they provide over conventional therapy is the ability to provide consistent treatment over longer periods of time. Patients with all levels of impairment can be treated based on the adaptive nature of the robots. These technologies can reduce the load on rehabilitation professionals and augment their ability to provide care to patients. Another benefit of these technologies is the added capability to collect vast amounts of data, track progress, and provide feedback to the patient and caregiver. This opens the door for other technological advances, such as those made in mobile health, machine learning, and telemedicine, to be incorporated into the rehabilitation engineering space and improve the quality of care.

### Potential robot-based areas of focus

While there are many areas that rehabilitation robotics span, we will briefly highlight a few that are relevant to designing neurorehabilitation strategies and considerations for applying these to people living with both HIV and stroke. These areas include robot-based biomarkers of motor impairment, motor learning, cognitive assessment and rehabilitation, and affordable rehabilitation robots. We discuss various open questions and potential research directions in each of these areas as they relate to the HIV-stroke population.

#### Robot-based biomarkers of motor impairment

The ability to quantify kinematic and dynamic measures of motor impairment is a key feature of robot-based systems, allowing for both higher resolution and reliability compared to clinical tests. There has been a lot of research into different metrics that are reflective of motor impairment [104, 106, 107]. The development of these metrics allows for assessment to be administered in a quicker manner and for progress to be tracked throughout the course of rehabilitation. Another benefit of robot-based biomarkers is the ability to potentially reduce the sample size needed to test a rehabilitation strategy, allowing for more efficient experiments [107]. In areas where trained rehabilitation professionals are in short supply or assessments are not feasible, having a robot assist in assessment and treatment can increase accessibility to quality treatment as well as reveal new information about areas that have been typically difficult places to gather data. Robot-based biomarkers of motor impairment have shown to be effective as it relates to stroke and has the potential to be useful for the HIV and HIV-stroke populations as well. However, the episodic nature of HIV causes patients to have variability as it relates to task performance, and how the episodic nature might affect motor recovery before and after stroke is an open question that could potentially be addressed by robot-based biomarkers.

#### Motor learning

Recovery of motor function is often seen as an extension of the motor learning process, consisting of motor adaptation, skill acquisition, and decision making [108]. Motor learning principles have been used to develop more effective rehabilitation strategies such as impairment-oriented training, constrained-induced movement therapy, electromyogram-triggered neuromuscular stimulation, robot-based therapy, and virtual-reality based rehabilitation [109]. Other strategies based on motor learning have also been explored, such as errorless learning or error augmentation [110, 111]. A typical motor learning experiment involving a robot consists of holding the end of a planar robotic arm and making reaching movements while the robot produces a perturbation force unknown to the subject that alters their trajectory [112]. A challenge to applying motor learning principles to robot-based rehabilitation is ensuring that actual learning rather than just motor adaptation is occurring [113].

Implicit and explicit learning are the two main methods of achieving motor outcomes. Explicit motor learning is defined as “learning which generates verbal knowledge of movement performance, involves cognitive stages within the learning process and depends on the involvement of working memory” while implicit learning “progresses with no or minimal increase in verbal knowledge of movement performance and without awareness” [114]. This implies that implicit learning involves the development of inherent habitual responses while explicit learning involves systematic processing of each step of the task [114]. Implicit and explicit learning have been studied in controlled laboratory environments, without a clear consensus of the value of one versus the other or which method is more effective in rehabilitation [115–119]. Some literature suggests that explicit feedback can interfere with the motor learning process in patients recovering from stroke and that implicit learning strategies may be more effective for patients with more cognitive deficits [120–122].

The combination of robotics and models of motor learning has resulted in the emergence of the computational neurorehabilitation field [123]. While there are many challenges associated with the field and dealing with an impaired population, grounding strategies in motor learning principles can lead to beneficial outcomes in the HIV-stroke population. Given the wide range of cognitive and motor impairments, identifying the best motor learning strategies under different conditions remains an open research question but, if addressed, can personalize and optimize recovery for this population.

#### Cognitive assessment and rehabilitation

Based on people who acquire brain injury including stroke, there is a need to expand the diversity of populations who can benefit from robot-based rehabilitation. A recent review of 120 rehabilitation robots shows that a majority of the treatment strategies are force-based or vision-based (virtual reality) systems using explicit motor learning strategies, and only a few robot therapy systems use implicit motor learning strategies such as error-augmentation control strategy [122, 124]. Research has shown that there is an association between cognition — particularly executive function and attention — and motor recovery, thus necessitating a focus on the cognitive aspects as well during rehabilitation [125]. Non-robot based strategies combining cognitive strategy and task-specific training demonstrated transfer of improvements to untrained activities and better performance compared to regular occupational therapy in stroke patients [126]. Cirstea and colleagues showed that successful motor intervention involving knowledge of performance feedback rather than knowledge of results led to motor and clinical improvements that were related to better memory, mental flexibility, and planning abilities [95].

However, robot-based strategies have the potential to be applied to the cognitive space. Bourke et al. used a robotic hit-and-avoid task to test rapid selection and generation of motor responses which involve cognitive and motor processes [127]. Additionally, robot-based measures have been shown to correlate with clinical measures in TBI patients [128]. Assessing cognitive performance can allow for novel rehabilitation applications, such as closed-loop control of cognitive load during a robot-assisted gait training task [129]. A better understanding of the cognitive aspects of impairment and how they affect motor recovery is important for designing rehabilitation strategies for the HIV-stroke population going forward, given the increased likelihood of joint cognitive and motor impairments. While neuropsychological and screening tests often separate the assessment of motor and cognitive domains, robot-based strategies are an opportunity to provide assessment and rehabilitation of tasks that involve both motor and cognitive domains.

#### Toward affordable rehabilitation robotics

The rapid development in the field of robot-assisted technologies in rehabilitation has opened the door for affordable solutions to take hold, making care more accessible [130]. Despite this, these technologies are not yet widely available even in HICs and thus may limit implementation and accessibility to such solutions in LMICs. Similarly, current rehabilitation robotics systems, while potentially cost-effective in the long run, require an initial amount of capital that may not be feasible for lower resource areas. The WHO guidelines state that cost-effective therapy solutions are those that cost less than three times the national gross domestic product for each respective country [131]. An example of this can be seen when Bustamante-Valles and colleagues were able to set up a robot-assisted rehabilitation gym in Mexico to supply care in an affordable and effective manner that allowed therapists to see more patients [130]. As the majority of people living with disabilities reside in LMICs, a more concerted effort to design cost-effective robot-based solutions for rehabilitation will increase the utility and application of such devices in LMICs, expanding the reach and scope of the rehabilitation robotics field.

### Potential non robot-based areas of focus

While rehabilitation robotics is a potential approach to designing neurorehabilitation strategies for the HIV-stroke population, it is not the only solution available. There has been development in other forms of rehabilitation that have focused on LMICs. These strategies can more readily address psychosocial and accessibility issues than the rehabilitation robotics field can in its current state. Two strategies in particular — community-based rehabilitation and home-based rehabilitation — have been particular areas of focus.

#### Community-based rehabilitation

In order to improve the quality of life for people with disabilities in LMICs and address some of the barriers, the WHO introduced the concept of community-based rehabilitation (CBR), which consists of programs that “are designed to meet the basic needs of people with disabilities, reduce poverty, and enable access to health, education, livelihood, and social opportunities” [132]. According to the WHO’s global disability action plan for 2014-2021, barriers that prevent access to rehabilitation, assistive technologies, and services for the disabled population in LMICs include high costs, insufficient number of trained professionals, absence of facilities and equipment, ineffective service models, and lack of integration and decentralization of services [14]. However, CBR programs have shown initial success in LMICs in increasing independence, self-esteem, and income [133].

In the context of HIV management, a study conducted in the United Kingdom and Canada demonstrated that community-based exercise programs are safe and can improve the quality of life of people living with HIV [134]. Benefits of community-based rehabilitation include increased social support, enhanced engagement in social activities, and reduced isolation and stigma associated with HIV [134]. A recent review of 24 studies showed that performing aerobic and resistive exercise is safe and can lead to improvements in cardio-respiratory fitness, strength, body composition and quality of life for adults with HIV [96]. CBR has also been tested in the stroke population and shown to be safe and effective [135, 136]. The initial CBR research in both HIV and stroke populations indicate that CBR has the potential to be applied in the HIV-stroke population. Other forms of community-based rehabilitation need to be tested beyond exercise-based programs, such as incorporating telemedicine, as well as the effectiveness of implementing these strategies in lower resource areas. The additional challenges posed by the HIV-stroke population and how those might change the approach of CBR strategies also need to be further researched.

#### Home-based rehabilitation

A component of CBR is home-based rehabilitation (HBR). HBR has been shown to be effective in HICs for chronic disease management and has the potential to be extended to LMICs [137]. A recent randomized controlled study in South Africa showed that a 16-week home-based rehabilitation program for people living with HIV carried out by community health care workers showed similar benefits to the standard of care [138]. HBR relies more on the patients themselves to drive rehabilitation, but this method can reduce costs by being based outside of institutions. Patients can still be observed by professionals either with follow ups or on home visits.

In the context of HIV-stroke care, CBR and HBR would allow patients to receive treatment in a more comfortable setting while avoiding some of the challenges presented with seeking institution-based treatment, such as high costs and traveling long distances. HBRs can be more easily implemented than CBRs, which require coordination across many different moving parts. However, some of the potential challenges of HBRs include generating support among policy makers, training sufficient workers, ensuring patient adherence, and translating the same successes seen in HICs to LMICs [137]. The lack of monitoring also makes assessing the true effects of HBRs a difficult task. A major challenge is the compatibility of HBR with other rehabilitation strategies that require equipment that people may not be able to afford. Further research into effective CBR and HBR strategies is needed, but they are a potential approach for designing rehabilitation strategies for the HIV-stroke population that can increase the accessibility to treatment.

## A potential approach for HIV-stroke neurorehabilitation

### Combining robot-assisted and community-based rehabilitation techniques

While both robot-assisted and community-based rehabilitation strategies address some of the challenges, neither of these approaches is suited perfectly for the HIV-stroke population. With robot-assisted rehabilitation, the social factors, such as the stigma associated with HIV, are not necessarily taken into account. In addition, given that rehabilitation robotics is mostly targeted at motor recovery but the HIV-stroke population will also present with varying degrees of cognitive deficits, the cognitive load of a task and its impact on task performance must also be taken into account. With CBR, there remains the challenge of reducing poverty, scaling up solutions, and promoting evidence-based practices. Mechanisms to track data and integrate services are also missing. In other words, there is still a need in LMICs for a solution rooted in accessibility, affordability, and analytics.

Combining the two approaches, however, could be a way to develop an effective, innovative form of neurorehabilitation. The strengths of each fill in the holes of the other. CBR provides a way to address the social aspects that are not met with rehabilitation robotics alone. The potential for scalable, affordable treatment and the ability to record data using robot-assisted rehabilitation would be a way to provide the quantitative analysis needed to promote the best evidence-based practices.

Current commercial rehabilitation robotic systems could be adapted to incorporate CBR-based methods[124, 139]. For example, the In Motion system (Bionik Labs) for upper limb rehabilitation and the variety of lower limb rehabilitation systems from Hocoma Inc. can be used in ways that promote increased health, livelihood, and social opportunities for the HIV-stroke population. The drawbacks of current commercial solutions are the high cost and scalability of these systems to LMICs. Thus, more innovation in this space is needed to be able to meet the functional, social, and emotional needs of the population.

### The Rehab CARES Gym

Our lab has designed a system called the Rehabilitation Community-Based Affordable Robot Exercise System (Rehab CARES) Gym, that is meant to provide robot-assisted rehabilitation in a community-based setting with the intention of deploying it in various LMICs through partnerships with local universities and health systems. We envision this system being based in primary care, tertiary care, or community centers. The current system is a compact robotic gym that provides affordable, game-based rehabilitation for the upper and lower limbs, based on concepts first tested in Mexico [130, 140]. Unlike existing rehabilitation systems that are bulky, expensive, and serve a single patient at a time, our setup enables one rehabilitation professional to treat multiple patients at a time in a more efficient manner [130]. It promotes a community-based approach by creating a fun and social therapy environment where patients can interact with each other, increasing their motivation to exercise and receive treatment. The unique aspects of this system include its modularity and adaptation of rehabilitation technologies that can be implemented in low-resource settings [140].

The system consists of various stations that serve different purposes. The passive stations consist of off-the-shelf rehabilitation equipment that provide patients with minor to moderate disability the capacity to improve functionality, with the ability to manually adjust the resistance. While these do not provide assistance, we have equipped them with sensors and motors to interface with games to adaptively adjust the resistance based on performance. The active station of the gym consists of a low-cost, single-degree-of-freedom adaptive haptic robot for upper limb rehabilitation called the Haptic TheraDrive [141]. This robot adjusts the amount of assistance based on the user’s performance in order to provide haptic feedback, allowing for people with severe impairments to interact with the system as well. Parallel bars and a sensorized walking platform for lower limb gait assessment and training are also part of the system. Together, the separate stations provide caregivers the ability to oversee multiple patients at once and provide patients access to consolidate different forms of rehabilitation in a single location. In the cases of cognitive impairment, we envision the tasks adapting the difficulty or cognitive load to the patient in a way that maintains a caregiver’s ability to oversee multiple patients at once, although what this would exactly look like is an open question. This way, the system may be adapting the robotic assistance in addition to the task itself to suit the patient’s motor and cognitive impairments. Each station is designed to collect assessment and performance data, which can be used to monitor progress and offer recommendations for a more personalized approach to rehabilitation. While the current configuration has three passive stations, one active station, and one gait station, the overall design of the system is modular in nature, meaning that the parts and combinations can be adjusted to meet the needs and resources of different areas. All of these stations would be integrated to allow for cooperative or competitive multiplayer games or for data to be collected for the same patient across different stations.

The Rehab CARES Gym’s data collection capabilities allow for experiments on robot-based biomarkers of motor and cognitive impairment as well as exploration of motor learning to be conducted. More research should focus on the best ways to measure motor and cognitive deficits with the system. One approach we have tried is assessing unilateral upper limb kinematics in both the impaired and less impaired limb sides using a variety of tasks that engage both motor and cognitive domains. Our hypothesis is that metrics exploring the relationship between the impaired and less impaired side could potentially be used to assess both cognitive and motor deficits across the stroke, HIV, and HIV-stroke populations [142]. This approach is supported by other recent work in stroke subjects [127]. Other avenues to explore include designing additional robot-based tasks that can jointly quantify a wider variety of cognitive and motor domains. Eventually, our goal is that these solutions can be implemented together in one system able to provide treatment for the impairments seen across the HIV-stroke spectrum.

The possible limitations and challenges of this solution vary depending on the context in which it is being implemented. In HICs, a challenge would be convincing high resource areas that have sufficient access to rehabilitation services to adopt such technologies. Therapists’ preference for interacting directly with their patients can slow the acceptance of robot-based solutions even if it provides similar benefits. Additionally, rehabilitation robot technologies are still considered experimental by many health insurance companies and are thus not reimbursed. In LMICs, additional social and cultural considerations may come into play, on top of other challenges such as powering the system, mobility of the system, and training to operate the system. Cost and resource constraints may also reduce some of the functionality of the system, making cost effectiveness analyses important [130].

## Conclusion

Developing relevant neurorehabilitation strategies is a critical component in the care and treatment of people living with the effects of both HIV and stroke. The long term physical, cognitive, and social effects of both conditions necessitate extensive monitoring, assessment, and treatment. The most relevant strategies will be ones that not only take into account the complex interactions occurring in the patient but also the cultural and economic considerations of their respective environment.

While there are many challenges posed by the HIV-stroke population, addressing them can benefit additional populations beyond just the HIV-stroke population to advance research in a variety of fields. It will require coordination between experts in various fields such as stroke, HIV, rehabilitation engineering, global health, and health care, among other areas. With a more concerted effort toward designing affordable rehabilitation robotics solutions and drawing on other rehabilitation strategies such as community-based rehabilitation, there is an opportunity to expand multiple fields in new and exciting directions.

## Abbreviations

AIDS: Acquired immunodeficiency syndrome
ANI: Asymptomatic neurocognitive impairment
ART: Antiretroviral therapy
CBR: Community-based rehabilitation
FA: Fractional anisotropy
HAD-HIV: Associated dementia
HAND-HIV: Associated neurocognitive disorder
HBR: Home-based rehabilitation
HDS: HIV dementia scale
HIC: High income country
HIV: Human immunodeficiency virus
IHDS: International HIV dementia scale
LMIC: Low and middle income country
MCD: Minor cognitive disorder
MoCA: Montreal cognitive assessment
MRI: Magnetic resonance imaging
Rehab CARES: Rehabilitation community-based affordable robot exercise system
TPA: Tissue plasminogen activator
WHO: World health organization
